# FDA approval of prademagene zamikeracel (Zevaskyn): advancing gene therapy for recessive dystrophic epidermolysis bullosa (RDEB) patients

**DOI:** 10.1097/MS9.0000000000004501

**Published:** 2025-12-02

**Authors:** Syeda Rabiah Shahid, Kamran Shahid, Maliha Khalid, Aminath Waafira

**Affiliations:** a Department of Medicine, Shaikh Khalifa Bin Zayed Al-Nahyan Medical and Dental College, Lahore, Pakistan; b Department of Medicine, Jinnah Sindh Medical University, Karachi, Pakistan; c School of Medicine, The Maldives National University, Malé, Maldives

*Dear Editor,*

On 29 April 2025, the U.S. Food and Drug Administration (FDA) approved Zevaskyn (prademagene zamikeracel) – a novel, first-of-its kind cell-based gene therapy – for Recessive Dystrophic Epidermolysis Bullosa (RDEB), transforming the treatment of a rare and debilitating genetic skin disorder^[1]^. This autologous, topical, genetically modified cell-based therapy serves as an epitome of the advancements in targeted modern medicine, offering a disease-modifying approach for a condition formerly managed with only palliative measures.

RDEB is an autosomal recessive rare disorder with an estimated prevalence of 3.5–20.4 per million people worldwide^[2]^. The underlying pathology of RDEB stems from mutations in the *COL7A1* gene which encodes collagen type VII – a critical component of structural fibrils that maintains dermal – epidermal cohesion and ensures skin integrity. Therefore, its absence results in the hallmark features of RDEB, including extreme skin fragility, recurrent blistering, chronic non-healing wounds, progressive scarring, and a heightened risk of cutaneous squamous cell carcinoma^[3]^.

The ensuing wounds are excruciatingly painful and can persist for years without healing. Despite the severity and predominantly infantile patient population of the disease, the current standard of care is solely supportive, primarily via protective bandaging and daily wound care. Thus, therapeutic regimens until now have failed to address the underlying defect or prevent future injuries^[4,5]^

Zevaskyn revolutionizes the therapeutic approach by delivering functional *COL7A1* into the patient’s own keratinocytes via an HSV-1-derived vector. These genetically modified cells are grown *ex-vivo* into graftable tissues, able to produce collagen type VII. Subsequent application of these grafts over the wounds restores epithelial integrity and promotes healing^[6]^.

**HIGHLIGHTS**

- ZEVASKYN (Prademagene zamikeracel) is the first FDA-approved autologous gene therapy for RDEB, targeting the *COL7A1* gene defect.
- A single topical application significantly improves wound healing and reduces pain, with durable effects lasting months to years.
- Despite clinical success and a favorable safety profile, the $3.1 million cost presents a major barrier to accessibility and equitable treatment.

This recent FDA approval follows positive outcomes evident in various clinical trials^[7]^, including the crucial phase 3 VIITAL study: a multi-center, randomized, intra-patient-controlled trial. The trial exhibited a greater than 50 percent rate of wound closure, as well as a marked reduction in pain scores from baseline within 6 months^[8]^. A single application of Zevaskyn – evidenced by the former trial results – claims to considerably enhance healing and reduce pain, thus offering a substantial benefit and durability of this intervention over existing therapies^[1]^.

Zevaskyn had a good safety index with minimal adverse events. Individuals involved in the trial had zero severe adverse events, with no evidence of immune-related symptoms. The most common negative effects were pruritus, wound infection, and pain at the injection site^[1,8]^. The infections were local and easily treated with topical or oral antibiotics, with no participants progressing to systemic involvement^[8]^. A long-term analysis of 18 patients treated with Zevaskyn, having a median follow-up period of 38.2 months, revealed no serious adverse reactions and no incidence of squamous cell carcinoma (SCC), as opposed to the SCC that was observed in 4 patients who had wound sites untreated with the therapy^[9]^. These findings represent an upward trajectory in the management of RDEB, which has, till now, been considered nigh-incurable.

Despite the promising prospects, the $3.1 million investment per treatment poses significant barriers to access, especially for the underinsured general population^[10]^. Although high, the cost is still relative to other gene-therapies like CRISPR-Cas9, which costs more than $2 million per dose without being specific to RDEB^[11]^. The pricing dilemma of advanced therapeutics has long been debated, yet government-level initiatives for subsidization and inclusion in insurance formularies are still warranted.

The FDA’s approval of Zevaskyn represents a major advancement, particularly in dermatological regenerative medicine and genetics. The immense potential of this therapy to modify therapeutic approaches is countered by the regulatory discourse surrounding the financial handicap, as well as obscurity of long-term safety, especially in the pediatric population. Therefore, further research, reanalyses of financial constraints and long-term surveillance are warranted in order to integrate its use in to clinical practice. Zevaskyn has not only offered hope to RDEB patients but has also paved the path for the future development of advanced disease-modifying therapeutics for debilitating inherited disorders. Our work is in line with the TITAN Guidelines on the need for transparency in AI use in healthcare.

## Data Availability

No data were generated for this manuscript.

## References

[1] Abeona Therapeutics Inc. [Internet]. U.S. FDAApproves ZEVASKYNTM (Prademagene Zamikeracel), the First and Only Cell-Based Gene Therapy for Patients with Recessive Dystrophic Epidermolysis Bullosa (RDEB). 2025.

[2] Eichstadt S Tang JY Solis DC. From Clinical phenotype to genotypic modelling: incidence and prevalence of Recessive Dystrophic Epidermolysis Bullosa (RDEB). Clin Cosmet Investig Dermatol 2019;12:933–42. 10.2147/CCID.S232547 PMC6935313 31920360

[3] Soro L Bartus C Purcell S. Recessive dystrophic epidermolysis bullosa: a review of disease pathogenesis and update on future therapies. J Clin Aesthetic Dermatol 2015;8:41. PMC4445895 26029334

[4] Rashidghamat E McGrath JA. Novel and emerging therapies in the treatment of recessive dystrophic epidermolysis bullosa. Intractable Rare Dis Res 2017;6:6–20. 28357176 10.5582/irdr.2017.01005 PMC5359356

[5] Hou P Agua ND Lwin SM. Innovations in the Treatment of Dystrophic Epidermolysis Bullosa (DEB): current Landscape and prospects. Ther Clin Risk Manag 2023;19:455–73. 37337559 10.2147/TCRM.S386923 PMC10277004

[6] MPR [Internet]. Zevaskyn Approved for Recessive Dystrophic Epidermolysis Bullosa. 2025.

[7] Eichstadt S Barriga M Ponakala A. Phase 1/2a clinical trial of gene-corrected autologous cell therapy for recessive dystrophic epidermolysis bullosa. JCI Insight 2019;4. 10.1172/jci.insight.130554 PMC6795403 31578311

[8] “Established safety profile.” ZEVASKYN™ (prademagene zamikeracel) Safety Profile. Abeona Therapeutics Inc. 2025. https://zevaskynhcp.com/safety/

[9] Fda approves Zevaskyn for rare, genetic skin disorder [Internet]. Accessed 1 May 2025.

[10] Ledford H. World first: ultra-powerful CRISPR treatment trialled in a person. Nature 2025;641:1083. 40389531 10.1038/d41586-025-01593-z

[11] Agha RA Mathew G Rashid R. Transparency In The reporting of Artificial INtelligence – the TITAN guideline. Premier J Sci 2025;10:100082.

